# LanthMS: A Computational Tool for the Structure Elucidation of Lanthipeptides from Tandem Mass Spectrometry Data

**DOI:** 10.2174/0109298665461951260413052350

**Published:** 2026-04-24

**Authors:** Lingyun Zhao, Wenya Zhao, Yujing Li, Yang Zhang, Yana Wang, Feiyan Zhang, Yingxue Feng, Liping Zhang, Hongwei Liu

**Affiliations:** 1 Institute of Biology, Hebei Academy of Sciences, Shijiazhuang, 050081, P.R. China;; 2 Main Crops Disease of Microbial Control Engineering Technology Research Center in Hebei Province, Shijiazhuang 050081, P.R. China;; 3 Hebei Jiaotong Vocational and Technical College, Shijiazhuang 050081, P.R. China

**Keywords:** Antimicrobial peptides, *Bacillus amyloliquefaciens*, bioinformatics, computational prediction, dehydration, post-translational modifications

## Abstract

**Introduction/Objective:**

Lanthipeptides are a class of ribosomally synthesized peptides with intricate ring structures, whose structural elucidation poses significant challenges. This study aimed to develop a computational tool named LanthMS to efficiently and accurately determine the topology of lanthipeptides directly from tandem Mass Spectrometry (MS/MS) data, thereby overcoming the limitations of conventional approaches in deciphering their dehydration and cyclization modifications.

**Methods:**

This study developed the specialized software LanthMS. The software exhaustively enumerates all possible lanthipeptide structures derived from given peptide sequences and assigns multidimensional scores by comprehensively comparing theoretical spectra against experimental MS/MS data, thereby predicting the most probable structures.

**Results:**

Using this approach, two novel lanthipeptides, amyA and amyC, were identified, from the *Bacillus amyloliquefaciens* WS-8 strain.

**Discussion:**

The LanthMS tool developed and validated in this study provides an automated solution for the structural elucidation of lanthipeptides. It not only significantly reduces the difficulty and subjectivity of manual interpretation but also deeply integrates computational structural prediction with experimental mass spectrometry data. This establishes a key technological framework for accelerating the discovery of lanthipeptides with novel activities and guiding their rational engineering.

**Conclusion:**

As a specialized *in silico* prediction tool, LanthMS substantially reduces the burden of manual interpretation, enhances the efficiency and accuracy of structural confirmation, and serves as a powerful engine for rapidly exploiting and engineering lanthipeptides with novel activities.

## 1 INTRODUCTION

Since the mid-20^th^ century discovery of antibiotics, their deployment has fundamentally transformed the management of bacterial infections [1-7]. However, widespread antibiotic misuse has precipitated a global public health crisis characterized by escalating antimicrobial resistance, underscoring an urgent need for novel therapeutic agents [8-16]. Lanthipeptides—a prominent subclass of ribosomally synthesized and post-translationally modified peptides (RiPPs) [17-22]—have emerged as compelling candidates owing to their characteristic thioether-bridged architectures, potent biological activities, and unique mechanisms of action, demonstrating efficacy against diverse pathogens including antimicrobial, antiviral, and immunomodulatory functions [22-24]. Exemplifying this class, nisin (a type I lanthipeptide) produced by *Lactococcus lactis* exhibits broad-spectrum antimicrobial activity [25-29]. Its designation as Generally Recognized As Safe (GRAS) has enabled decades of successful application as a biopreservative across dairy, meat, and beverage industries in over 50 countries, where it effectively controls spoilage organisms such as *Listeria monocytogenes* and *Clostridium* spp [30-32]. Building upon this established safety profile and mechanistic understanding, current research is actively extending nisin's therapeutic relevance beyond food preservation toward clinical applications, collectively highlighting the versatile potential of lanthipeptides as multifunctional molecular agents [33].

The unique bioactivities of lanthipeptides stem directly from their structural complexity, characterized by the presence of non-proteinogenic amino acids lanthionine (Lan) and/or methyllanthionine (MeLan) [34, 35]. These signature motifs arise through thioether bridges connecting either two alaninyl residues (derived from cysteine and a dehydrated serine) or an aminobutyryl residue with an alaninyl residue (derived from cysteine and a dehydrated threonine). These interlocking cyclic topologies constitute foundational structural determinants that confer exceptional resistance to proteolytic degradation and extreme pH/temperature conditions, thereby enabling biological function under physiological stress [36-38]. Post-translational modifications—notably enzymatic dehydration followed by regiospecific cyclization—represent the core biosynthetic machinery. Crucially catalyzed by lanthipeptide synthetases (LanM/LanC), target serine (Ser) and threonine (Thr) residues in the core peptide undergo dehydration to form dehydroalanine (Dha) and dehydrobutyrine (Dhb). Subsequent intramolecular Michael addition of cysteine (Cys) thiols to the electrophilic α,β-unsaturated bonds of Dha/Dhb generates the stereoselective thioether crosslinks, ultimately yielding the Lan/MeLan cyclic architectures essential for bioactive conformation [39-42].

The programmed dehydration of Ser/Thr residues and subsequent thioether cyclization are hallmark biosynthetic steps that confer unparalleled chemical diversity and topological heterogeneity to lanthipeptides. Paradoxically, this intricate structural complexity poses fundamental challenges for modern analytical methodologies, particularly for Mass Spectrometry (MS)-based structural elucidation. Conventional peptide identification relies on predictable fragmentation patterns during tandem MS (MS/MS), where Collision-Induced Dissociation (CID) or Higher-energy Collisional Dissociation (HCD) typically generates diagnostic N-terminal b-ions and C-terminal y-ions from linear peptide backbones [43-46]. Sequential mass differences among these ions enable straightforward amino acid sequencing. In stark contrast, lanthipeptides adopt diverse ring topologies (linear, nested, or overlapping configurations) resulting from combinatorial dehydration/cyclization events. A single precursor peptide can yield hundreds of isobaric isomers due to variable dehydration sites and ring connectivities [47, 48]. Critically, the conformationally constrained lanthipeptide scaffolds exhibit non-stochastic fragmentation dynamics: the disruption of each thioether bridge necessitates at least two consecutive backbone cleavages. Consequently, canonical b/y-ion series are suppressed, replaced by low-abundance internal fragments and non-sequential cross-ring fragments with obscure spectral patterns [49-53]. These intrinsic characteristics render mainstream proteomic pipelines—predicated on gene-encoded linear sequences and predictable PTM behavior in databases (*e.g*., Mascot, Sequest, X!Tandem)—fundamentally inadequate for lanthipeptide structure determination [54-56]. The urgent need for dedicated computational tools capable of deciphering this topological complexity is unequivocal.

To systematically address the discovery of peptides that originate from unannotated genomic regions, for instance, those encoded by small Open-Reading Frames (sORFs) or non-coding RNAs, the field has witnessed the emergence and success of integrated peptidogenomics and proteogenomics pipelines [57-60]. These approaches fundamentally shift the paradigm from searching static, canonical protein databases to constructing dynamic, sample-specific databases derived directly from genomic and transcriptomic data, such as RNA-seq. A landmark demonstration of this strategy is the development of an integrated peptidogenomic pipeline for the large-scale discovery of non-conventional peptides in maize and Arabidopsis [61-64]. Similar integrated frameworks, such as those designed for neoantigen discovery in oncology or for streamlined omics data analysis, further underscore the power of converging multi-omics data for peptide discovery [65-69]. While these pipelines have revolutionized the discovery of linear or modestly modified peptides from novel genomic loci, the uniquely complex, branched, cyclic topologies of heavily post-translationally modified peptides, such as lanthipeptides, pose a distinct challenge that often falls outside their design scope.

It is important to clarify that the PTMs themselves (dehydration and cyclization) are enzymatic processes catalyzed by LanM synthetases and do not occur in the gas phase during MS analysis. Mass spectrometry detects the stable, modified products of these cellular processes. Furthermore, while integrated ‘peptidogenomics’ pipelines have advanced the discovery of ribosomally synthesized natural products, the specialized topology of lanthipeptides demands dedicated computational solutions.

To bridge this methodological gap, LanthMS, a computational framework for high-accuracy lanthipeptide structural elucidation was developed. This approach synergizes precursor peptide sequences with experimental MS/MS spectra through an automated deconvolution pipeline, eliminating error-prone manual interpretation. Core to this innovation is a topology enumeration engine that exhaustively models all feasible dehydration/isomerization patterns with their resultant ring connectivities, coupled with a multi-parametric scoring algorithm—derived from theoretical-experimental spectral comparisons—that quantifies matches based on fragmentation propensity, ion intensity correlations, and diagnostic neutral loss signatures. By enabling end-to-end topological elucidation directly from MS data, LanthMS establishes a pivotal technological framework for accelerating the discovery and bioengineering of novel lanthipeptides.

## 2 MATERIALS AND METHODS

### 2.1 Software Architecture and Core Algorithmic Workflow

**Figure 1:**
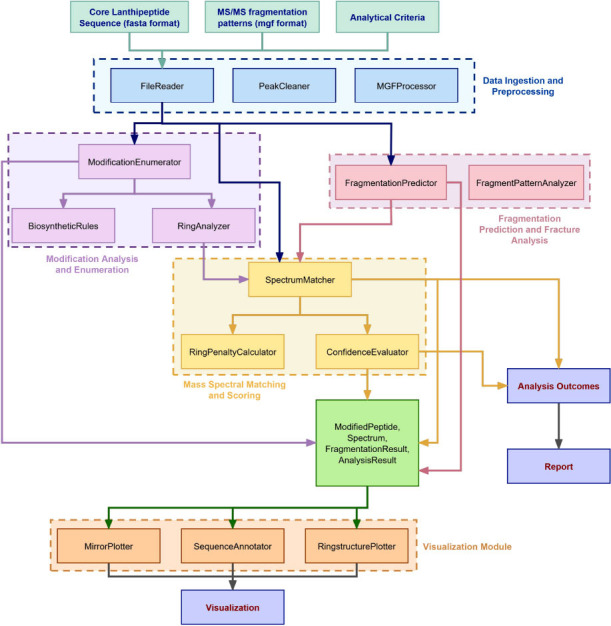
The architecture of the LanthMS. This flowchart illustrates the step-by-step workflow of the LanthMS software. Specifically, it begins by taking in a peptide sequence and mass spectrometry data, processes them through five core computational modules, and ultimately outputs the predicted structure of a lanthipeptide. This figure provides a clear overview of the automated pipeline that replaces manual interpretation.

LanthMS is a specialized bioinformatics platform for structural characterization of lanthipeptides containing dehydration modifications and cyclization motifs. The system processes peptide sequences in FASTA format and tandem mass spectrometry data in MGF format (Mascot generic format) through an integrated analytical pipeline that culminates in comprehensive visualization outputs. The workflow sequentially executes five core computational modules (Figure **1**): initial data acquisition and preprocessing handles input normalization; subsequent modification analysis systematically enumerates all possible lanthipeptide structural variants; fragmentation prediction then performs *in silico* cleavage analysis; mass spectral matching subsequently scores enumerated structures against experimental spectra; ultimately, the visualization module renders interactive analytical representations to facilitate structural interpretation.

### 2.2 Mass Calculation System

LanthMS employs a precision mass calculation framework to ensure accurate matching between theoretical and experimental masses. The theoretical mass of unmodified peptides is calculated as follows in Equation 1:


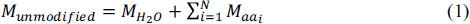


where:

*Maa_i_* - the monoisotopic mass of the i-th amino acid residue

*N* - peptide length (number of residues)

*MH_2_O* - the molecular mass of water (18.010565 Da, accounting for terminal dehydration).

For modified peptides, the theoretical mass incorporates modifications, as defined in Equation 2:


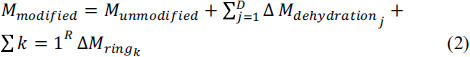


where:

*D* - the dehydration modification count

Δ*Mdehydration_j_* = -18.010565 Da

*R* - the cyclization modification events

Δ*Mring_k_* = 0 Da, due to internal bond formation during lanthionine/methyllanthionine generation.

Experimental precursor neutral masses is calculated using Equation 3 as follows:


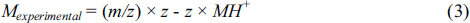


where:

*m/z* - the precursor mass-to-charge ratio

*z* - the charge state

*MH^+^* - the proton mass (1.007276 Da)

Mass errors were computed as Equation 4:


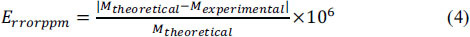


### 2.3 Aggregate Match Score

LanthMS employs a composite scoring system that quantitatively evaluates multiple dimensions of fragment ion matching, with scoring metrics comprising an aggregate match score and a confidence score.

The aggregate match score is calculated as Equation 5:


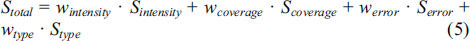


with term weights configured as follows: *w_intensity_* = 0.4, *w_coverage_* = 0.3, *w_error_* = 0.2, *w_type_* = 0.1 (Table **S1**).

Intensity score is calculated as Equation 6:


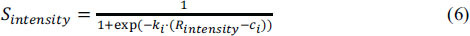


where:

- the ratio of matched fragment intensity to total experimental intensity

*k_i_* - the slope factor (default = 15)

*c_i_* - the center point (default = 0.1)

Coverage score is calculated as Equation 7:


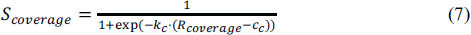


where:


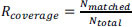
 - matched fragments-to-total theoretical fragments ratio,

*k_c_* - the slope factor (default = 10)

*c_c_* - the center point (default = 0.3)

Mass error score is calculated as Equation 8:


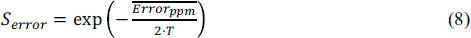


where:


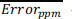
 - the mean ppm error across all matched fragments

*T* - the mass tolerance threshold (default = 20 ppm)

Note: This fragment mass tolerance (default = 20 ppm) is applied during the precise scoring of matched ions. A much wider precursor mass tolerance (default: 6000 ppm) is used in the initial software workflow to ensure comprehensive enumeration of candidate structures, accounting for the significant mass shifts induced by multiple dehydrations.

Fragment-type score is calculated as Equation 9:


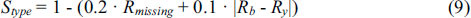


where:

*R_missing_* - the proportion of missing critical ions (*e.g*., b-, y-type).

*R_b_* and *R_y_* - specify the relative abundances of matched b and y ions, respectively.

|*R_b_* - *R_y_*| - quantifies the b/y ion abundance imbalance.

### 2.4 Confidence Score

The confidence score incorporates cyclic topology features alongside intensity, coverage, mass error, and fragment type considerations. This composite metric is computed as Equation 10:


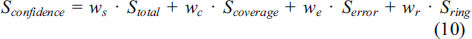


using the coefficient assignment: *w_s_* = 0.6, *w_c_* = 0.2, *w_e_* = 0.1, *w_r_* = 0.1 (Table **S1**).

Cyclic bond cleavage penalty is quantified by Equation 11:


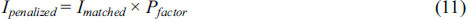


where:

*I_penalized_* - the penalized fragment intensity

*I_matched_* - the original matched intensity

*P_factor_* - the penalty factor (default = 0.7)

Confidence classification thresholds are defined as follows in Equation 12-15 (Table **S1**):


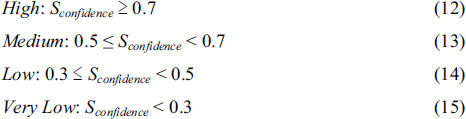


### 2.5 Software Implementation and Workflow

The computational scripts of LanthMS were developed in Python and executed on Windows 10/11 64-bit operating systems. The LanthMS software features an intuitive tabbed interface comprising five functional components: a menu bar supporting essential operations including new analysis initiation, result loading, and help documentation access; an analysis tab housing file selection controls and parameter configuration fields; a results tab dedicated to analytical outcomes visualization; a logging window displaying real-time processing metadata and progress updates; and a status bar providing system notifications (Figure **S1**).

Users initiate analysis by importing two input files: peptide sequences in standard FASTA format (with entries beginning with a ‘>’ symbol followed by sequence identifiers and corresponding amino acid sequences) and mass spectrometry data in MGF format (containing precursor m/z values, charge states, and fragmentation peaks). Prior to execution, users designate an output directory and configure critical parameters, including mass tolerance threshold (default: 6000 ppm), maximum allowable cyclic structures per analyte (default: 5), maximum dehydro modifications (default: 10), minimum confidence level (options: low/medium/high), and enable visualization output (Table **S1**). Following parameterization, the analysis is triggered *via* the ‘Run Analysis’ button, with generated visuals subsequently available for inspection and export from the results interface.

### 2.6 Experimental Samples and Mass Spectrometric Validation

#### 2.6.1 Bioinformatic Identification of Lanthipeptide Biosynthetic Gene Clusters Using B. amyloliquefaciens WS-8

Putative class II lanthipeptide biosynthetic gene clusters within the genome of *B. amyloliquefaciens* WS-8 were identified bioinformatically using BAGEL3 [70], a specialized platform for bacteriocin and RiPP (ribosomally synthesized and post-translationally modified peptide) gene cluster prediction. The complete genome sequence was analyzed with BAGEL3 using the default parameters (minimal cluster score: 0.6; HMM database version 2022). Potential gene clusters associated with class II lanthipeptide biosynthesis were identified and analyzed. Structural characterization of the core peptide regions employed leader peptide prediction algorithms, with cleavage sites recognized based on double glycine motifs identified with a probability threshold set to ≥ 85%.

#### 2.6.2 Construction of Lanthipeptide Heterologous Expression Vector

Genomic analysis of *B. amyloliquefaciens* WS-8 revealed a biosynthetic locus encoding LanM A/LanM B modification enzymes, the LanT150 transporter (containing a peptidase domain), and precursor peptides amyA, amyB and amyC. Codon optimization of these genes (*lanM A*, *lanM B*, *lanT150*, *amyA, amyB, amyC*) for *Escherichia coli* expression was conducted using ExpOptimizer (https://www.novopro.cn/tools/codon-optimization.html; accessed on 14 February 2026) with GC content constrained to 45–55% and codon adaptation index (CAI) ≥ 0.85. Synthesized genes (NovoPro, Shanghai) were cloned into expression vectors *via* Gibson assembly: (a) *lanM A* and *lanM B* inserted into pACYCDuet-1, yielding pACYCDuet-LanM A/ LanM B; (b) *lanT150* ligated into pCDFDuet-1, generating pCDFDuet-1-LanT150; (c) *amyA, amyB, amyC* subcloned into pET-28a(+) to produce pET-28a-A/B/C. Antibiotic resistance screening was employed to identify the transformed bacteria by selecting transformants on LB agar plates supplemented with kanamycin (50 µg/mL), chloramphenicol (25 µg/mL), and spectinomycin (50 µg/mL), followed by overnight incubation at 37^o^C. Colonies exhibiting resistance to all three antibiotics were considered primary positive clones. The vectors were subsequently validated by sequencing (Sangon Biotech, Shanghai).

#### 2.6.3 Transformation and Identification of Co-expression Strains

Co-expression strains were generated by transforming *E. coli* BL21(DE3) with the three plasmids encoding precursor peptides (pET-28a-A/B/C), LanM A/ LanM B synthases (pACYCDuet-LanM A/ LanM B), and the LanT150 transporter (pCDFDuet-1-LanT150) *via* the transformation method, as outlined in Table **1**. Control strains received the empty pET-28a vector, along with synthase/transporter plasmids. Transformants were selected on LB agar containing antibiotics (kanamycin, chloramphenicol, spectinomycin) for overnight incubation. Putative co-expressing colonies were verified by (a) simultaneous resistance to all three antibiotics and (b) colony PCR amplification using Taq DNA polymerase with vector-specific primers.

#### 2.6.4 Expression and Purification of Lanthipeptides

Heterologous expression was employed to induce the co-expression of precursor peptides, synthetases, and the transporter protein peptidase domain. The induction was achieved using 0.2 mM IPTG at 25°C for 20 h. Post-incubation, cells were pelleted *via* centrifugation at 8000 rpm for 20 min.

A methanol extraction procedure was carried out by treating a 100 mL fermentation broth with a 10:1 ratio of methanol to cells for 4 h. The supernatant was filtered through a 0.22 µm organic filter membrane into a sample vial for further analysis. High-performance liquid chromatography (HPLC) was employed to identify distinct peaks, utilizing a Shim-pack GIST C18 column (20 mm × 250 mm, 5 μm, Shimadzu) with a detection wavelength of 204 nm. The mobile phase A comprised ultrapure water with 0.1% TFA, while mobile phase B consisted of HPLC-grade acetonitrile with 0.1% TFA.

A gradient elution technique with a flow rate of 1 mL/min. The elution program for HPLC detection is presented as followed: 10% B for 3 min, 10%-35% B linear gradient for elution for 10 min, 35%-50% B linear gradient for elution for 30 min, 50%-100% B linear gradient for elution for 10 min, 100% B isovolumic for elution for 10 min, 100%-10% B linear gradient for elution for 5 min, 10% B isovolumic for elution for 10 min. Peaks showing deviations from the control were collected.

#### 2.6.5 LC-QTOF-MS/MS Analyses

LC-MS/MS analyses were conducted using a UHPLC system (ExionLC AD, AB SCIEX, USA) with an ACQUITY UPLC HSS T3 C18 column (2.1 mm × 100 mm, 1.7 μm, Waters) coupled to a quadruple time-of-flight mass spectrometer (TripleTOF 5600+, AB SCIEX, USA). The column temperature was maintained at 30°C. Mobile phase A was acetonitrile containing 0.1% (volume ratio) formic acid, and B was HPLC-grade water containing 0.1% (volume ratio) formic acid for positive (ESI+) modes. The flow rate was 0.3 mL/min and the gradient was set as follows: 0-1 min: 5% B, 1-16 min: 5% B to 100% B, 16-18 min: 100% B, 18-18.1 min: 100% B to 5% B, and 18.1-23 min: 5% B. The injection volume was 2 μL.

Data acquisition was performed in Information-Dependent Acquisition (IDA) mode. The source parameters were set as follows: ion source gas 1 (GAS1), 35 psi; curtain gas (CUR), 30 psi; temperature (TEM), 550°C; declustering potential (DP), 80 V; collision energy, 35 V; and ion spray voltage floating (ISVF), +5500 V for positive mode. The TOF MS scan parameters were set as follows: mass range, 400-1600 Da; accumulation time, 0.15 s/spectra. The product ion scan parameters were set as follows: mass range, 100-1650 Da; accumulation time, 0.03 s/spectra.

## 3 RESULTS

### 3.1 Analyzing Lanthipeptide Synthesis Gene Cluster

**Figure 2:**
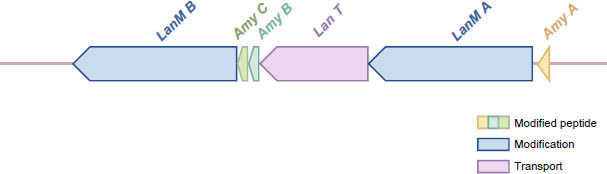
Diagram of lanthipeptide synthesis gene cluster from B. amyloliquefaciens WS-8. This genetic map identifies the set of genes responsible for producing novel lanthipeptides in the WS-8 bacterial strain. Key genes include those encoding the precursor peptides (*AmyA, AmyB, AmyC*), the modification enzymes (LanM A/ B), and the transporter (LanT). The discovery of this cluster is the starting point for finding the new peptides amyA and amyC.

Utilizing the bioinformatics tool BAGEL 3, the biosynthetic pathway of antimicrobial compounds in the genome of *B. amyloliquefaciens* WS-8 was analyzed. Sequence alignment studies indicated the existence of a gene cluster associated with the biosynthesis of class II lanthipeptides (Figure **2**).

Within this gene cluster, three genes were successfully identified, namely *amyA*, *amyB*, and *amyC*, encoding precursor peptides for lanthipeptides. Additionally, two synthase genes, *LanMA* and *LanMB*, were identified, responsible for catalyzing modifications on the lanthipeptides. Moreover, a transport protein gene, *LanT*, encoding a peptidase-domain-containing protein, was also identified.

Through sequence alignments of the three putative novel lanthipeptide precursors, conserved regions were observed in these precursor peptide genes. Notably, a high level of nucleotidesequence similarity, up to 85.88%, was observed when comparing the *amyB* and *amyC* sequences. The significant homology between *amyB* and *amyC* was further supported by their close proximity within the gene cluster (Figure **S2**).

### 3.2 Construction of Heterologous Expression Strains

To improve the expression of the target gene in *E. coli*, codon optimization of the synthase gene was carried out (Table **S2**). The optimized gene sequence of *LanMA* exhibited 76.15% nucleotide identity to the original sequence (Figure **S3**), while the optimized gene sequence of *LanM B* showed 75.81% nucleotide identity to the original sequence (Figure **S4**). Furthermore, the optimized gene sequence of *LanT150* displayed 77.04% nucleotide identity to the original sequence (Figure **S5**).

Co-transformation of *Escherichia coli* BL21(DE3) with three plasmids yielded eight co-transformant strains (Table **1**): PAT (empty vector control encoding LanM A synthetase and LanT150 transporter), PBT (empty vector control encoding LanM B synthetase and LanT150 transporter), AAT (precursor peptide pET-28a-A, LanM A synthetase, LanT150 transporter), ABT (precursor peptide pET-28a-A, LanM B synthetase, LanT150 transporter), BAT (precursor peptide pET-28a-B, LanM A synthetase, LanT150 transporter), BBT (precursor peptide pET-28a-B, LanM B synthetase, LanT150 transporter), CAT (precursor peptide pET-28a-C, LanM A synthetase, LanT150 transporter), and CBT (precursor peptide pET-28a-C, LanM B synthetase, LanT150 transporter). Positive transformants identified by triple antibiotic resistance selection were confirmed *via* PCR amplification. Agarose gel electrophoresis of the amplified fragments showed products matching the expected amplicon sizes, verifying the successful construction of each coexpression strain.

### 3.3 Purification of the Mature Lanthipeptides

**Figure 3:**
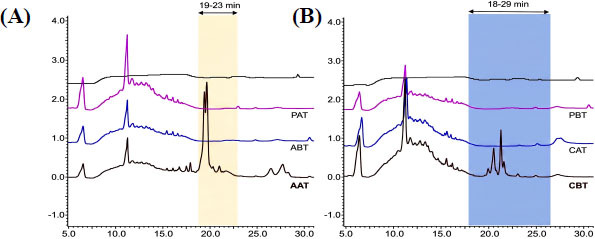
Expression results of co-expressed strain heterologous expression (**A**) AAT, from top to bottom: methanol control, PAT, ABT, AAT. (**B**) CBT, from top to bottom: methanol control, PBT, CAT, CBT. These HPLC chromatograms act like ‘chemical fingerprints’ to confirm successful production of the target lanthipeptides. The distinct peaks in panels **A** (for *amyA*) and **B** (for *amyC*) indicate that these peptides were only produced when the correct combination of precursor peptide and modifying enzyme, namely the AAT construct for *amyA* and the CBT construct for *amyC*, was present in the bacterial host, confirming specific enzyme activity. (Despite moderate peak intensity, the subsequent MS/MS analysis yielded high-quality fragmentation spectra suitable for structural elucidation).

The HPLC analysis of heterologous expression products from all co-expressed strains revealed prominent characteristic peaks for AAT and CBT only. In contrast, the remaining co-expression systems and the control strains PAT and PBT did not exhibit any noteworthy characteristic peaks. The lanthipeptide precursor peptide amyA underwent modification and processing by the LanMA synthetase, with co-expression of strain AAT. Analysis *via* HPLC chromatogram indicated the elution of the mature lanthipeptide amyA within the 19-23 min timeframe (Figure **3A**). The lanthipeptide precursor peptide amyC underwent modification and processing by the LanM B synthetase, with co-expression of strain CBT. Analysis *via* HPLC chromatogram revealed the elution of the mature lanthipeptide amyC within the 18-29 min timeframe (Figure **3B**). The remaining unexpressed co-strains may be attributed to the synthetase's stringent specificity, resulting in failure to accurately identify and modify the precursor peptide.

### 3.4 Structural Identification of *amyA*

**Figure 4:**
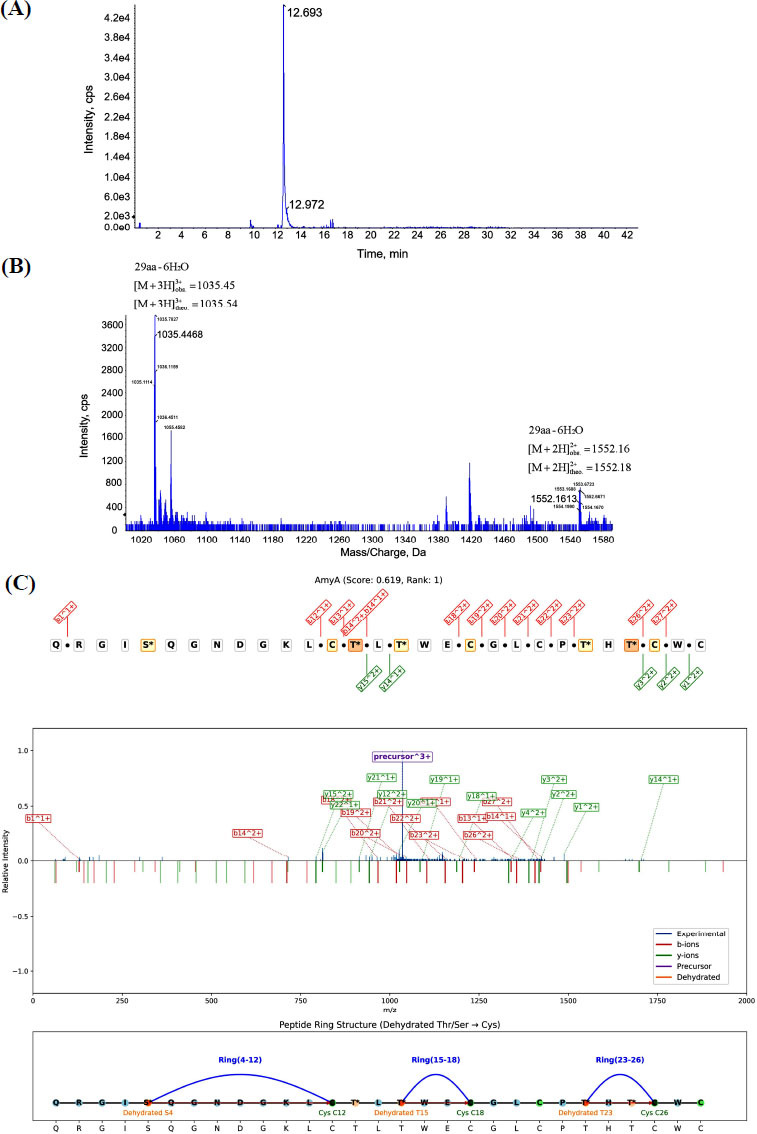
Purification and identification of amyA. (**A**) The High-Performance Liquid Chromatography (HPLC) chromatogram for amyA. (**B**) Primary mass spectrum of HPLC-purified amyA. (**C**) The highest-scoring LanthMS prediction for amyA. This figure presents the experimental and computational evidence for the structure of the novel lanthipeptide amyA. Panel **A** shows its purification profile, Panel **B** confirms its molecular weight, and Panel **C** visually summarizes LanthMS’s prediction of its precise chemical structure, including the locations of dehydrated amino acids and the rings formed by thioether bridges.

The heterologous expression product *amyA* from the co-expressed strain AAT was purified using HPLC, followed by structural identification using QTOF MS/MS. Based on the primary mass spectrum (Figure **4A** and **B**), a precursor ion with m/z 1035.45 was selected for secondary tandem mass spectrometry analysis.

Tandem mass spectrometry analysis of the lanthipeptide amyA revealed a core peptide comprising 29 amino acids. Computational analysis using LanthMS software indicated the occurrence of five dehydration events during amyA’s post-translational modification, specifically targeting serine at position 5 (Ser5), threonine at position 14 (Thr14), threonine at position 16 (Thr16), threonine at position 24 (Thr24), and threonine at position 26 (Thr26). Further, the top-ranked LanthMS prediction identified three thioether bridges: between Ser5 and Cys13, Thr16 and Cys19, and Thr24 and Cys27 (Figure **4C**). Notably, analysis of the top ten LanthMS predictions confirmed identical dehydration sites across all results (Ser5, Thr14, Thr16, Thr24, Thr26). However, alternative thioether ring formations were plausible, including potential linkages between Thr26 and Cys29; Thr14 and Cys22; or Thr24 and Cys27 (Figure **S6** and **S7**).

### 3.5 Structural Identification of *amyC*

**Figure 5:**
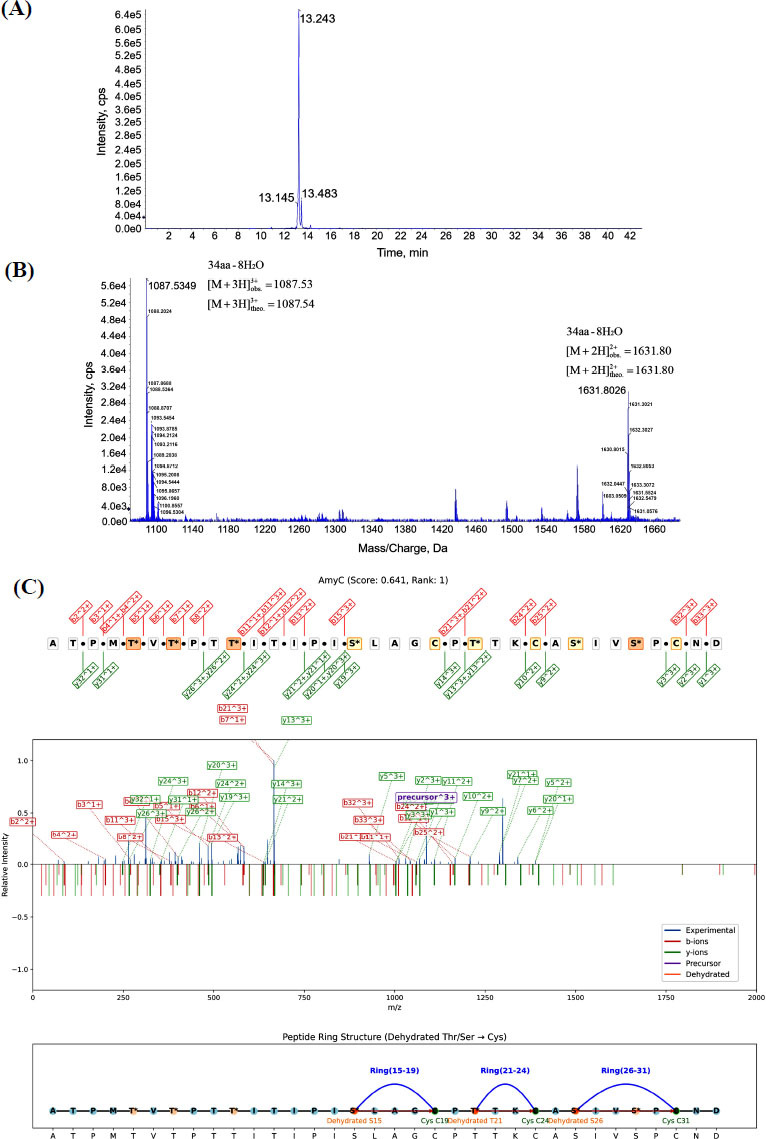
Purification and identification of amyC. (**A**) The High-Performance Liquid Chromatography (HPLC) chromatogram for amyC. (**B**) Primary mass spectrum of HPLC-purified amyC. (**C**) The highest-scoring LanthMS prediction for amyC. This figure presents the experimental and computational evidence for the structure of the second novel lanthipeptide, amyC. Panel **A** shows its purification profile, Panel **B** confirms its molecular weight, and Panel **C** visually summarizes LanthMS’s prediction of its chemical structure. Notably, the predicted pattern of thioether bridges in amyC is distinct from that of amyA, highlighting the structural diversity achievable within this peptide family.

Structural characterization of the heterologously expressed lanthipeptide amyC from the co-expression strain CBT was performed. Purification *via* HPLC followed by QTOF-MS/MS analysis identified the precursor ion at m/z 1087.53 (Figure **5A** and **B**). Tandem mass spectrometry analysis of the lanthipeptide amyC established its 34-residue core peptide structure. LanthMS software analysis revealed seven dehydration events during post-translational modification, with concomitant formation of three thioether bridges: between Ser16 and Cys20, Thr22 and Cys25, Ser27 and Cys32. The top-scoring prediction model localized dehydration sites at Thr5, Thr7, Thr10, Ser16, Thr22, Ser27, and Ser30 (Figure **5C**). Alternative dehydration sites at Thr9 and Thr12 were identified among the ten highest-ranking predictions (Figure **S8**, **S9**).

### 3.6 Amino Acid Profile of amyA and amyC

**Figure 6:**
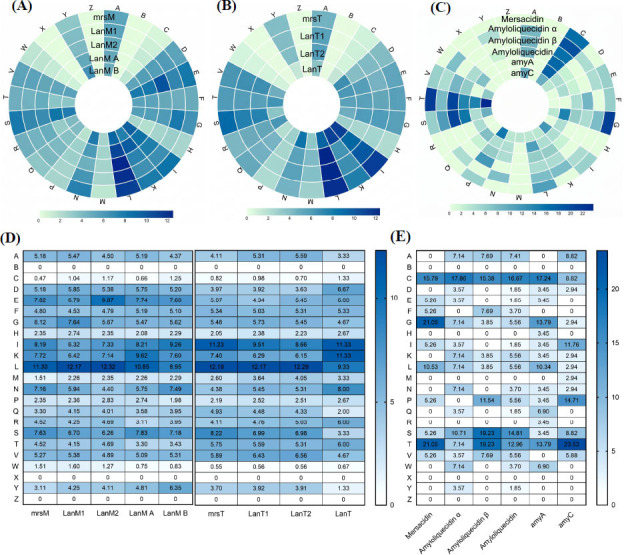
Amino acid profile of amyA and amyC. (**A**) Amino acid profile of LanM. (**B**) Amino acid profile of LanT. (**C**) Amino acid profile of lanthipeptides. (**D**) Amino acid composition of LanM and LanT. (**E**) Amino acid composition of lanthipeptides. This set of charts compares the amino acid composition of the newly discovered peptides and their associated enzymes with known relatives. It highlights both shared features and unique characteristics of amyA and amyC, providing insights into their potential functional and evolutionary relationships within the lanthipeptide family.

The amino acid sequences of LanM, LanT, and core peptides from amyA and amyC, alongside other class II lanthipeptides such as Mersacidin and Amyloliquecidin, were examined (Figure **6**). The amino acid composition of LanM proteins across these lanthipeptides exhibited notable similarities. In MrsM (LanM of Mersacidin), LanM1 (LanM of Amyloliquecidin α), LanM2 (LanM of Amyloliquecidin β), and LanM A (LanM of amyA), leucine (L) is the most abundant amino acid. In contrast, LanM B (LanM of amyC) is characterized by a predominance of isoleucine (I), followed by leucine (L). For the lantipeptides mrsT (LanT of Mersacidin), LanT1 (LanT of Amyloliquecidin α), and LanT2 (LanT of Amyloliquecidin β), leucine (L) amino acids are also the most prevalent; however, in LanT (LanT of amyA and amyC), isoleucine (I) and lysine (K) amino acids are most abundant, followed by leucine (L). Mersacidin has high proportions of glycine (G), threonine (T), and cysteine (C). Amyloliquecidin exhibits substantial proportions of cysteine (C), serine (S), and threonine (T). AmyA is distinguished by high levels of cysteine (C), glycine (G), and threonine (T), whereas amyC is marked by high proportions of threonine (T), proline (P), and isoleucine (I).

## 4 DISCUSSION

LanthMS integrates an exhaustive enumeration approach with a multi-parametric weighted scoring strategy, enabling automated and accurate prediction of lanthipeptide structures featuring complex dehydration and cyclization patterns. Its efficacy was demonstrated by the identification of two novel lanthipeptides, *amyA* and *amyC*, from *B. amyloliquefaciens* WS-8. As the structure elucidation platform explicitly tailored for lanthipeptides characterized by their dehydration-cyclization cores, its exhaustive strategy overcomes the ambiguity inherent in structural assignment by comprehensively capturing the target topology within the solution space. Crucially, the intelligent scoring system advances beyond conventional methodologies reliant on fragment ion count or low-ppm error matching. Instead, it implements a comprehensive weighted evaluation algorithm incorporating peak intensity, sequence coverage, mass error, fragment ion types, and ring cleavage patterns. This multi-dimensional framework significantly enhances prediction accuracy and, critically, the resolution capacity for intricate cyclic topologies. The platform exhibits substantial practical utility: by fully automating the interpretation workflow, it drastically reduces the time and manual effort required by traditional approaches. For instance, manual interpretation of lanthipeptide structures can take from several hours to days. In contrast, the entire LanthMS workflow, from data input to final output, requires only a few minutes in total, as its automated analysis typically completes in less than a minute. A key innovation is the provision of the top ten rank-ordered candidate structures rather than a single solution. This presents researchers with a comparable hypothetical space for evaluation—a design that acknowledges the ambient uncertainty in bioinformatic analysis and effectively addresses the need for flexible interpretation in practical research scenarios.

Nevertheless, LanthMS exhibits several critical limitations: it relies solely on the input core peptide sequence of lanthipeptides for structural enumeration, and the current version supports only dehydrations (Dha/Dhb) and thioether cyclization, rendering it incompatible with other PTMs such as phosphorylation or glycosylation. Future development could extend the enumeration engine to incorporate these modifications. Furthermore, its algorithm fails to incorporate steric hindrance effects from ring conformations, potentially leading to incomplete simulation of noncanonical cleavage patterns and requiring high-quality MS/MS spectra to ensure reliability. Applying LanthMS directly to complex *in vivo* samples (*e.g*., microbial culture extracts) poses additional challenges, including high matrix interference and the low abundance of target lanthipeptides, which require robust prefractionation and highly sensitive instrumentation prior to analysis.

The *Bacillus* genus is well recognized in contemporary research literature for its prolific capacity to produce diverse secondary metabolites, making it a prime resource for novel bioactive compound discovery [71-75]. This study centers on representative species within this genus, with the research trajectory exemplified by the seminal characterization of the lanthipeptide Mersacidin from *Bacillus* sp. HIL Y-85, 54728 by Fehlhaber and Kogler in 1992 [76], followed by the annotation of Amylolysin 3 in *B. amyloliquefaciens* GA1 by Arias *et al*. in 2013 [77]. Building upon this established context, systematic genome mining in this work unveiled a novel lanthipeptide biosynthetic gene cluster in *B. amyloliquefaciens* WS-8. This cluster comprises three precursor peptide genes (amyA, amyB, amyC) along with two synthetase genes (lanMA and lanM B) responsible for post-translational modifications. The synergistic application of MSMS and the LanthMS algorithm enabled a systematic elucidation of the modification mechanisms. LanM A modifies the amyA precursor peptide, undergoing five dehydration events and forming three thioether rings to yield the mature peptide amyA. Correspondingly, LanM B catalyzes the dehydration and cyclization of the *amyC* precursor, involving seven dehydration events and the subsequent formation of three thioether rings to produce the mature peptide *amyC*. It is noteworthy that both mature products ultimately undergo processing mediated by cleavage *via* the transporter LanT.

Collectively, this study establishes an integrative framework combining genome mining with the innovative LanthMS algorithm to systematically decipher the biosynthetic logic of novel lanthipeptides in *B. amyloliquefaciens* WS-8. As the first fully automated platform explicitly designed for dehydration-cyclization core structures, LanthMS’s multidimensional weighted scoring strategy has resolved the complex topologies of *amyA* and *amyC*.

## CONCLUSION

As a groundbreaking tool, LanthMS overcomes the critical ambiguities in MS/MS interpretation of complex lanthipeptide structures by exhaustively enumerating all potential modification/cross-linking patterns and employing a tailor-made multiparameter scoring scheme optimized for their fragmentation behavior. Its successful application, leading to the identification of two novel antimicrobial lanthipeptides, *amyA* and *amyC*, from *B. amyloliquefaciens* WS-8, effectively validates the method’s feasibility and accuracy. The availability of LanthMS provides crucial support for the discovery, structural characterization, and engineering of lanthipeptides.

## AUTHORS’ CONTRIBUTIONS

The authors confirm their contributions to the paper as follows: HL contributed to study conception and design; HL, LZ, and WZ carried out data collection; HL, LZ, WZ, YL, YZ, YW, FZ, YF, and LZ performed the analysis and interpretation of the results; and LZ and WZ prepared the draft manuscript. All authors reviewed the results and approved the final version of the manuscript.

## Figures and Tables

**Table 1:** Transformation system of combined expression strains.

**Transformation System**	**Strain**	**Abbr.**
pET-28a/pACYCDuet-LanM A/ pCDFDuet-1-LanT150	*E. coli* BL21(DE3)	PAT
pET-28a/pACYCDuet-LanM B/ pCDFDuet-1-LanT150	*E. coli* BL21(DE3)	PBT
pET-28a-A/pACYCDuet-LanM A/ pCDFDuet-1-LanT150	*E. coli* BL21(DE3)	AAT
pET-28a-A/pACYCDuet-LanM B/ pCDFDuet-1-LanT150	*E. coli* BL21(DE3)	ABT
pET-28a-B/pACYCDuet-LanM A/ pCDFDuet-1-LanT150	*E. coli* BL21(DE3)	BAT
pET-28a-B/pACYCDuet-LanM B/ pCDFDuet-1-LanT150	*E. coli* BL21(DE3)	BBT
pET-28a-C/pACYCDuet-LanM A /pCDFDuet-1-LanT150	*E. coli* BL21(DE3)	CAT
pET-28a-C/pACYCDuet-LanM B/ pCDFDuet-1-LanT150	*E. coli* BL21(DE3)	CBT

## Data Availability

All data generated or analyzed during this study are included in this published article. The LanthMS software tool, including its source code, user manual, example input files (FASTA and MGF formats), and a minimal reproducible workflow script, is publicly available on GitHub: https://github.com/lhwei1987/LanthMS.git.

## LIST OF ABBREVIATIONS

AMPs: Antimicrobial Peptides
RiPP: Post-Translationally Modified Peptide
HPLC: High-Performance Liquid Chromatography
IDA: Information-Dependent Acquisition
GAS1: Ion Source Gas 1
CUR: Curtain Gas
TEM: Temperature
DP: Declustering Potential
ISVF: Ion Spray Voltage Floating
