# Supplementary material for: LanthMS: A Computational Tool for the Structure Elucidation of Lanthipeptides from Tandem Mass Spectrometry Data
Source: Protein Pept Lett. 2026 Apr 24;33(2):439–53. doi: 10.2174/0109298665461951260413052350 (PMC13172255; doi:10.2174/0109298665461951260413052350)
Supplement: Supplementary file 1 [file PPL-33-2-439_SD1.pdf]

SUPPLEMENTARY MATERIAL

LanthMS: A Computational Tool for the Structure Elucidation of Lanthipeptides from Tandem Mass Spectrometry Data

Lingyun Zhao[1,3,#], Wenya Zhao[1,3,#], Yujing Li[2,#], Yang Zhang[1,3], Yana Wang[1,3], Feiyan Zhang[1,3], Yingxue Feng[1,3], Liping Zhang[1,3,*] and Hongwei Liu[1,3,*]

[1]Institute of Biology, Hebei Academy of Science, Shijiazhuang 050081, P.R. China; [2]Hebei Jiaotong Vocational and Technical College, Shijiazhuang 050081, P.R. China; [3]Main Crops Disease of Microbial Control Engineering Technology Research Center in Hebei Province, Shijiazhuang 050081, P.R. China

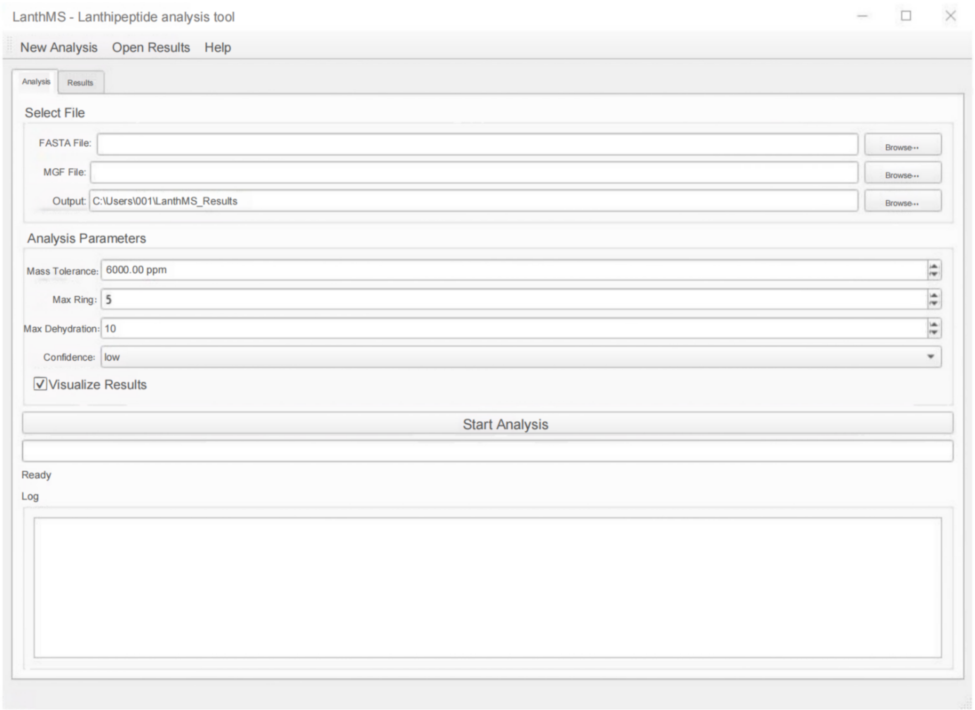

Figure S1. Operational interface design workflow of LanthMS. This figure shows the user interface layout of the LanthMS software, including the menu bar, analysis tab, and results window, and depicts the user workflow for file upload, parameter setting, and prediction result visualization.

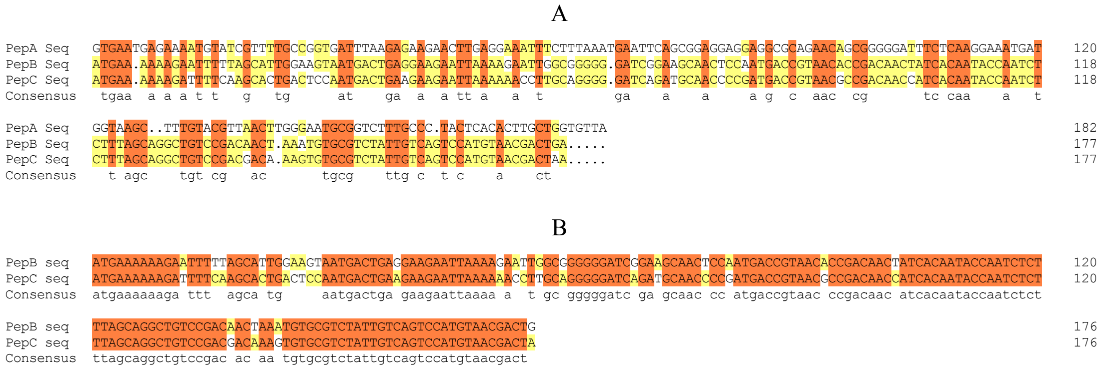

Figure S2. Comparison of propeptide DNA sequences. (A) Comparison of DNA sequences for *amyA*, *amyB*, and *amyC.* (B) Comparison of DNA sequences between *amyB* and *amyC*. This figure visually demonstrates the similarity among the three precursor peptide genes (*amyA*, *amyB*, *amyC*) through DNA sequence alignment. Panel B, in particular, shows the high degree of similarity between amyB and amyC, explaining their close proximity in the gene cluster and their potential evolutionary relationship.

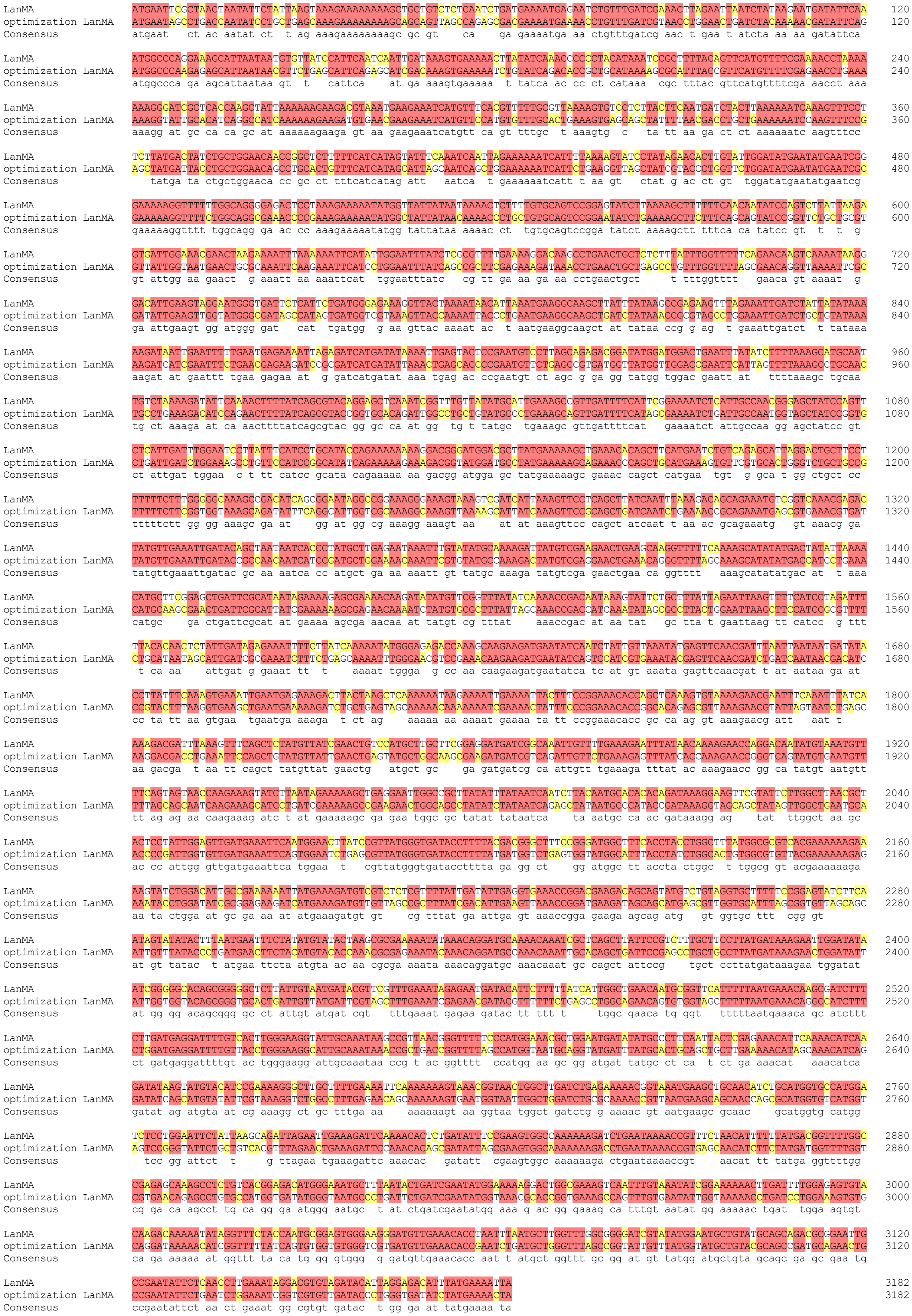

Figure S3. Alignment of nucleotide sequences of the optimized and original *LanMA* genes. This sequence alignment shows the 'codon optimization' performed on the *LanMA* synthetase gene for better expression in the engineering host. It displays the alignment between the optimized and original sequences, a key step for successful heterologous expression.

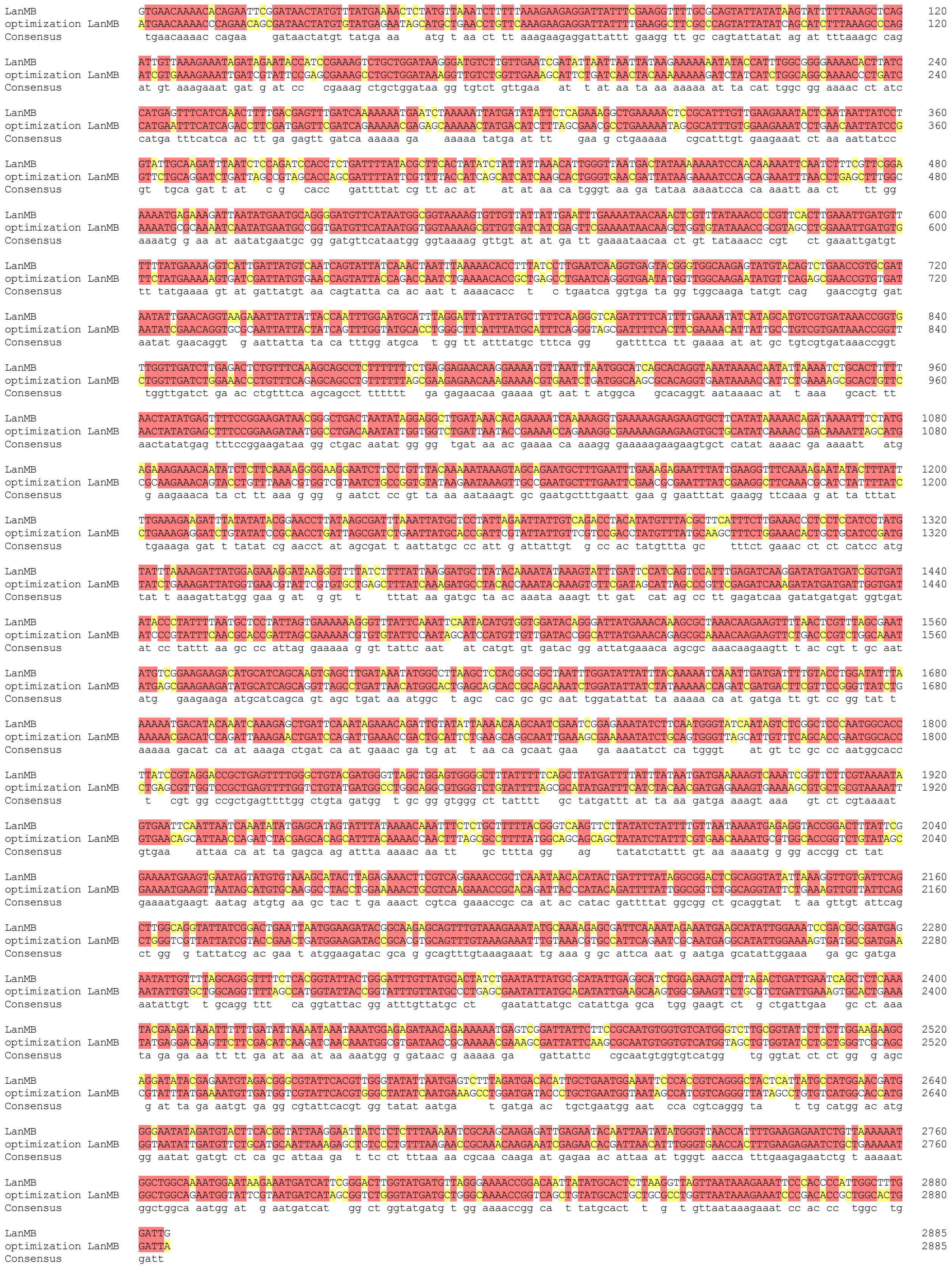

Figure S4. Alignment of nucleotide sequences of the optimized and original *LanM B* genes. This sequence alignment shows the 'codon optimization' performed on the *LanM B* synthetase gene to improve expression in the engineered host. It displays the alignment between the optimized and original sequences, a key step for successful heterologous expression.

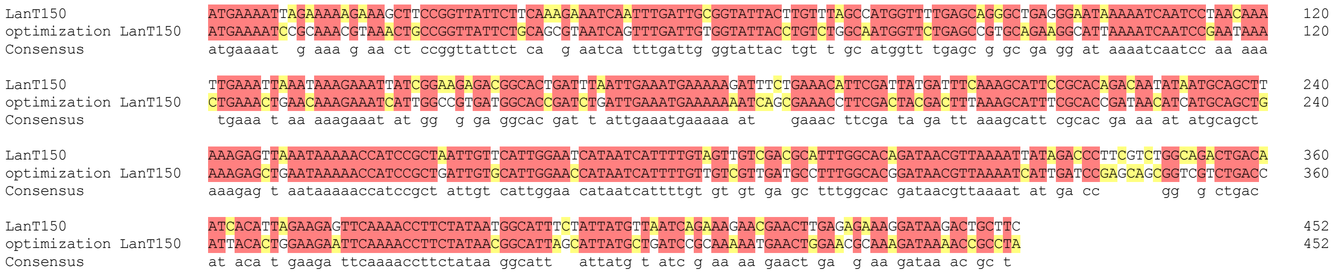

Figure S5. Alignment of nucleotide of the optimized and original *LanT150* genes. This sequence alignment shows the ‘codon optimization’ performed on the *LanT150* synthetase gene for better expression in the engineering host. It displays the alignment between the optimized and original sequences, a key step for successful heterologous expression.

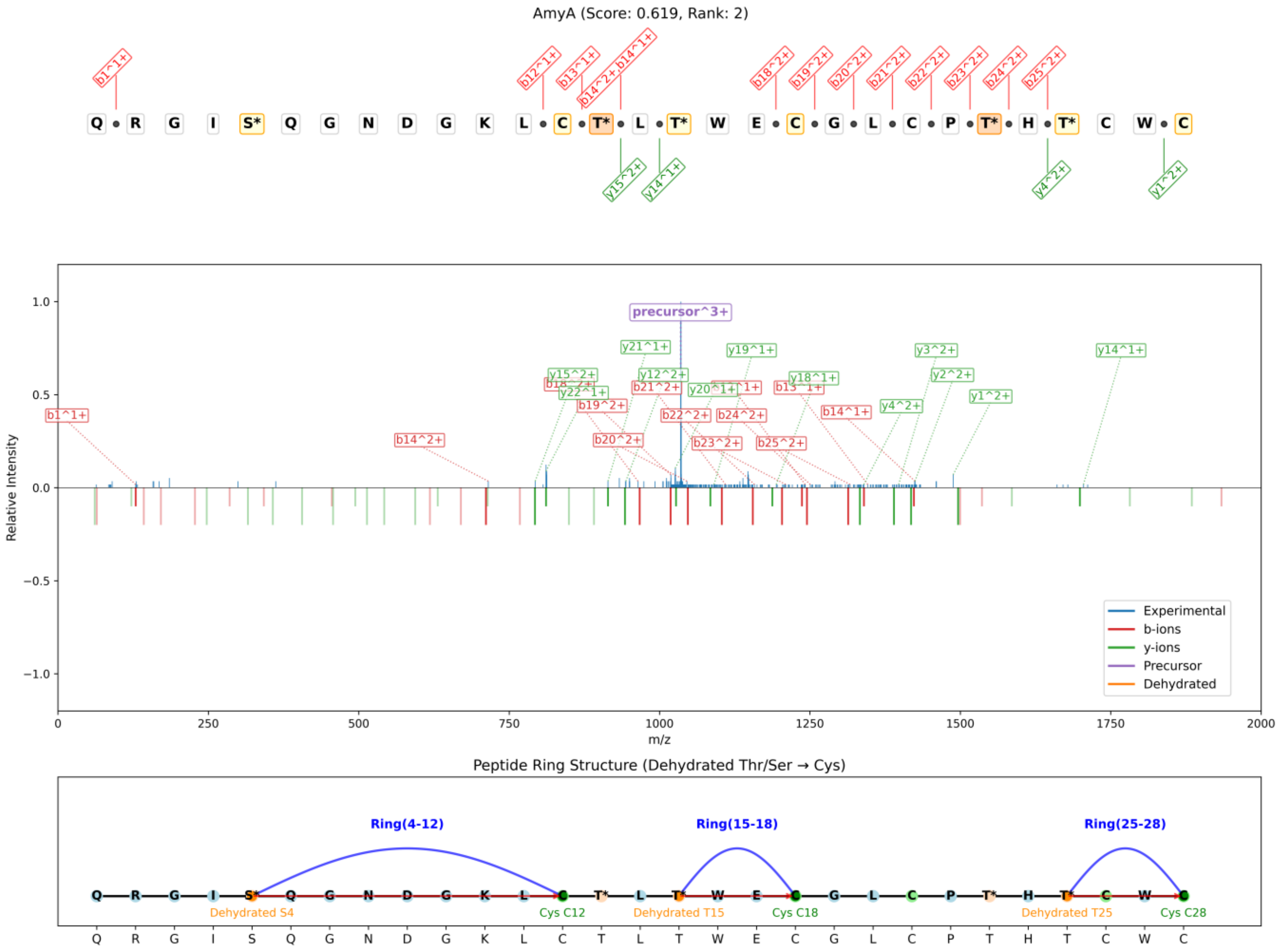

Figure S6. The second-highest-scoring LanthMS prediction result for amyA. This figure shows the second-highest-scoring candidate model predicted by LanthMS for the structure of amyA. Comparing it with the top model (Figure 4C) demonstrates the inherent uncertainty in deciphering complex cyclic topologies, offering researchers alternative structural possibilities for consideration.

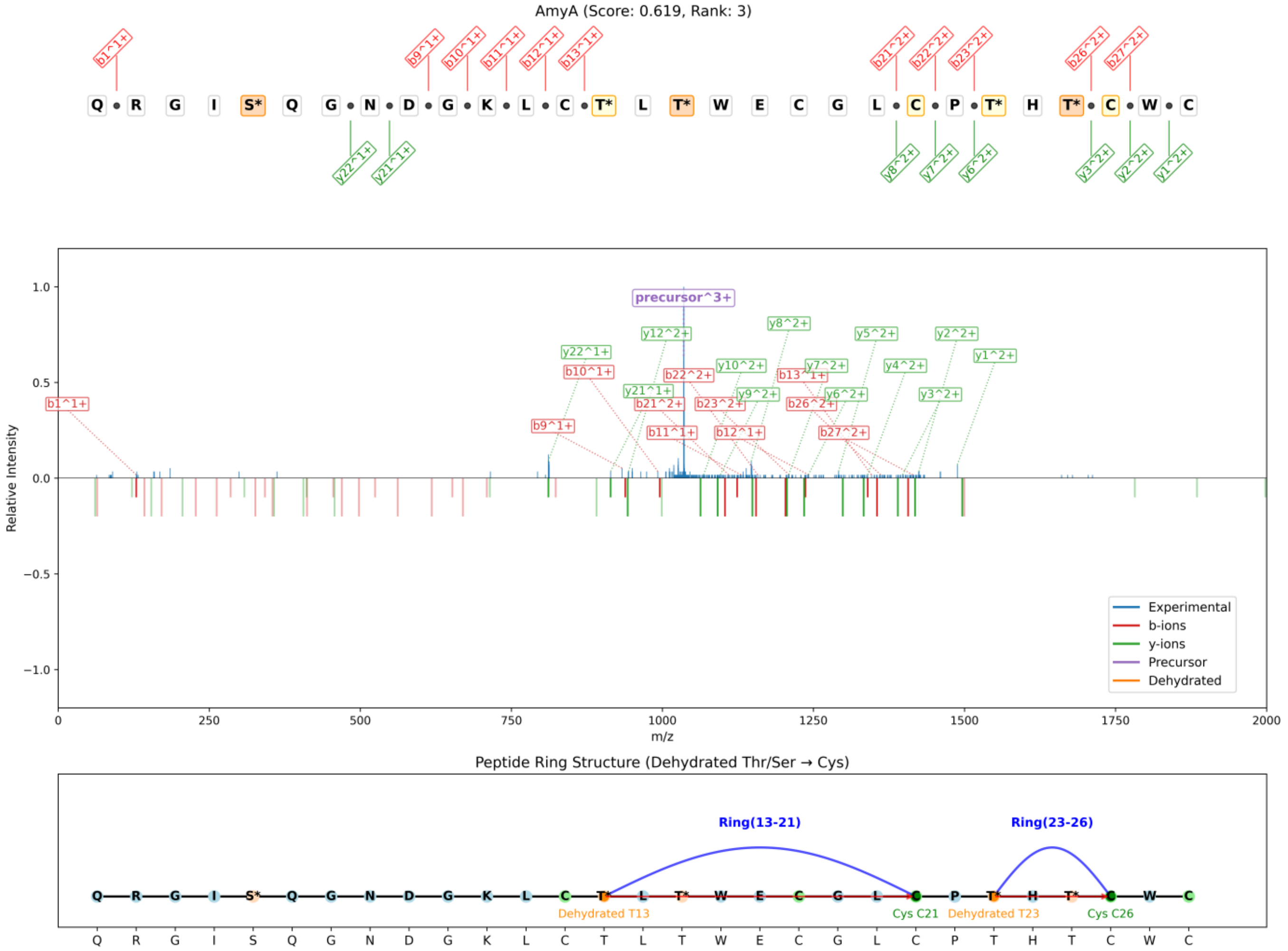

Figure S7. The third-highest-scoring LanthMS prediction result for amyA. This figure shows the third-highest-scoring candidate model predicted by LanthMS for the structure of amyA. Comparing it with the top model (Figure 4C) demonstrates the reasonable uncertainty inherent in deciphering complex cyclic topologies, providing researchers with alternative structural possibilities for consideration.

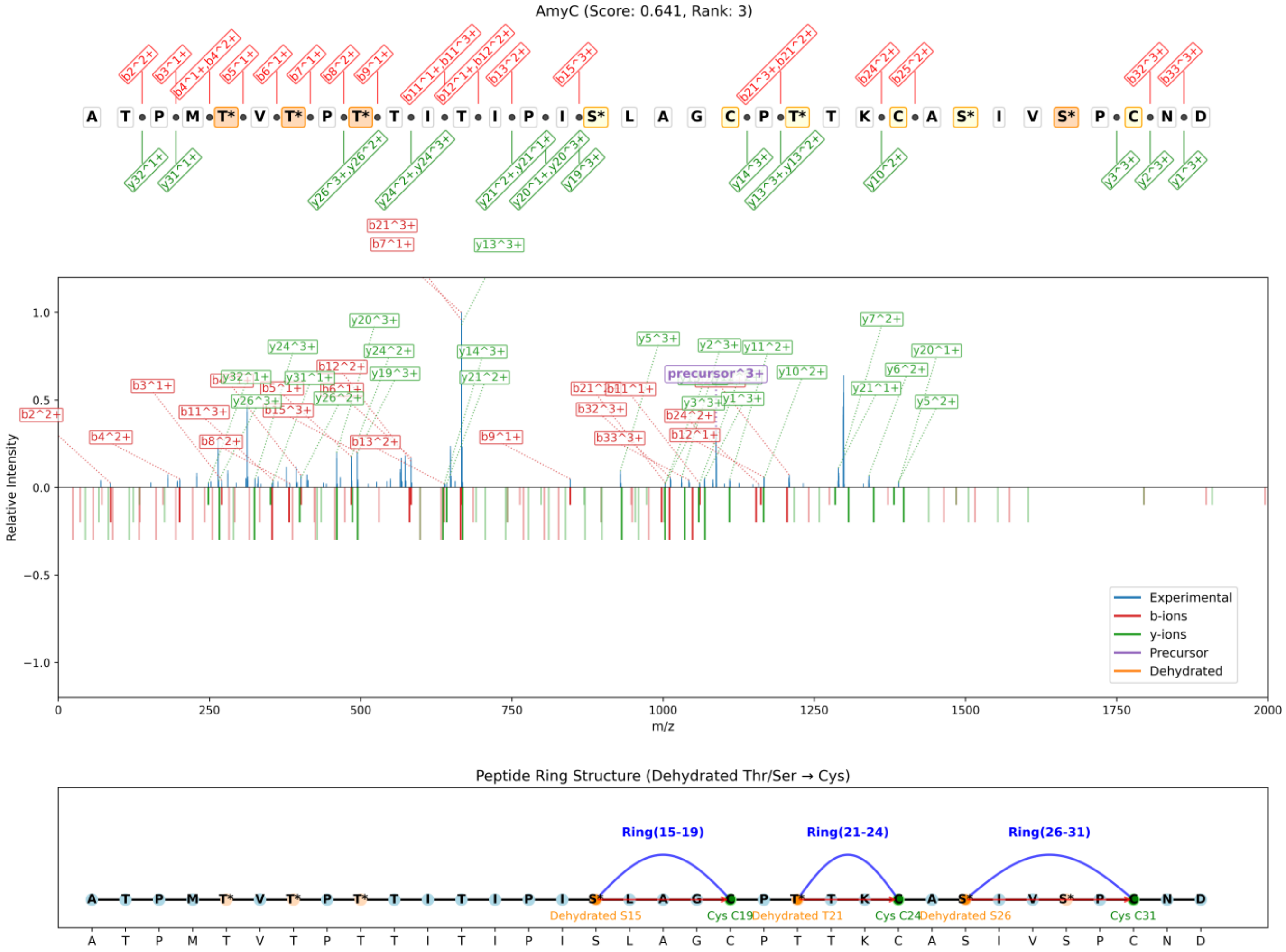

Figure S8. The second-highest-scoring LanthMS prediction result for amyC. This figure shows the second-highest-scoring candidate model predicted by LanthMS for the structure of amyC. Comparing it with the top model (Figure 5C) demonstrates the reasonable uncertainty inherent in deciphering complex cyclic topologies, providing researchers with alternative structural possibilities for consideration.

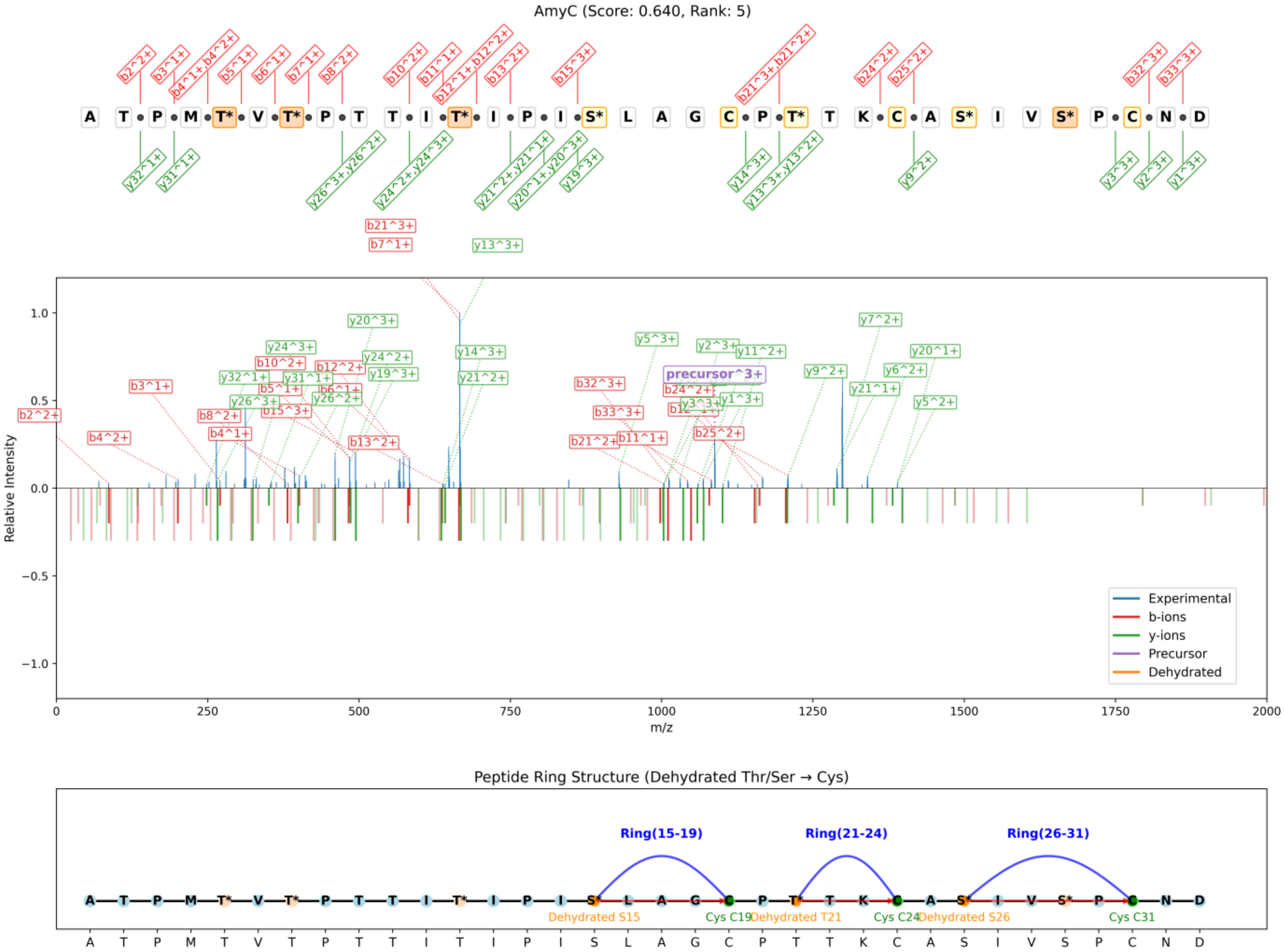

Figure S9. The third-highest-scoring LanthMS prediction result for amyC. This figure shows the third-highest-scoring candidate model predicted by LanthMS for the structure of amyC. Comparing it with the top model (Figure 5C) demonstrates the reasonable uncertainty inherent in deciphering complex cyclic topologies, providing researchers with alternative structural possibilities for consideration.

Table S1. Key parameters and default settings of the LanthMS workflow.

Parameter	Symbol/Name	Default Value	Purpose/Applied Stage
Precursor mass tolerance	-	6000 ppm	Initial filtering during candidate structure enumeration.
Fragment mass tolerance	T	20 ppm	Precision matching in the mass error score.
Max cyclic structures	-	5	Maximum number of top cyclic variants to output per peptide.
Max dehydro modifications	-	10	Maximum allowed dehydration events per peptide.
Intensity score weight (aggregate)	$w_{intensity}$	0.4	Weight of intensity correlation in aggregate score.
Coverage score weight (aggregate)	$w_{coverage}$	0.3	Weight of sequence coverage in aggregate score.
Mass error score weight (aggregate)	w_{error}	0.2	Weight of mass accuracy in aggregate score.
Fragment type score weight (aggregate)	w_{type}	0.1	Weight of ion type balance in aggregate score.
Aggregate match score weight	w_s	0.6	Weight of aggregate match score in confidence score.
Coverage score weight (confidence)	w_c	0.2	Weight of sequence coverage in confidence score.
Mass error score weight (confidence)	w_e	0.1	Weight of mass accuracy in confidence score.
Ring score weight (confidence)	w_r	0.1	Weight of ring penalty in confidence score.
Confidence - High threshold	-	0.7	Minimum score for classification as ‘High confidence’.
Confidence-Medium threshold	-	0.5	Minimum score for classification as ‘Medium confidence’.
Confidence - Low threshold	-	0.3	Minimum score for classification as ‘Low confidence’.

Table S2. Nucleotide sequence identity between the original and optimized genes.

Gene	Nucleotide identity (%)
LanM A	75.2%
LanM B	81.6%
LanT150	92.3%
